# Accumulation and transformation of inorganic and organic arsenic in rice and role of thiol-complexation to restrict their translocation to shoot

**DOI:** 10.1038/srep40522

**Published:** 2017-01-17

**Authors:** Seema Mishra, Jürgen Mattusch, Rainer Wennrich

**Affiliations:** 1 UFZ – Helmholtz Centre for Environmental Research, Department of Analytical Chemistry, Permoserstr. 15, D-04318 Leipzig, Germany; 2 CSIR-National Botanical Research Institute, Plant Ecology & Environmental Science Division, Rana Pratap Marg, Lucknow 226 001 (U.P.), India

## Abstract

Environmental contamination of arsenic (As) and its accumulation in rice (*Oryza sativa* L.) is of serious human health concern. *In planta* speciation of As is an important tool to understand As metabolism in plants. In the present study, we investigated root to shoot As translocation and speciation in rice exposed to inorganic and methylated As. Arsenate (As^V^) and methylarsonate (MA^V^) were efficiently reduced to arsenite (As^III^) and MA^III^, respectively in rice root and shoot but no trivalent form of dimethylarsinate (DMA^V^) was detected. Further, up to 48 and 83% of root As in As^V^ and MA^V^ exposed plants, respectively were complexed with various thiols showing up to 20 and 16 As species, respectively. Several mixed As- and MA-complexes with hydroxymethyl-phytochelatin, DesGly-phytochelatin, hydroxymethyl-GSH and cysteine were identified in rice. Despite high complexation in roots, more As was translocated to shoots in MA^V^ exposed plants than As^V^, with shoot/root As transfer factor being in order DMA^V^ > MA^V^ > As^V^. Moreover, in shoots 78% MA^III^ and 71% As^III^ were present as weakly bound species which is alarming, as MA^III^ has been found to be more cytotoxic than As^III^ for human and it could also be an important factor inducing straighthead (spikelet sterility disorder) in rice.

Arsenic (As) is ubiquitously present, considered as a non-essential metalloid for plants and animals, and poses serious health hazards to humans. High levels of As in soil and water have been reported around the world through geothermal, mining and industrial activities, agricultural applications and contaminated ground water^1^,^2^. Ground water of many countries, particularly in the Indian subcontinent, are naturally highly enriched with As and posing toxicity through drinking water and food chain contamination^3^,^4^. Agricultural fields are the net sink for thousands of tons of As being transferred each year through contaminated irrigation water in these areas^5^,^6^. The typical As concentration in soil solution of paddy fields varies from 0.01 to 3 μM, however, as high as 33 μM As has been reported in a paddy field irrigated with As-laden ground water^7^,^8^. Further, a significant yield loss has been reported in the crops grown in As contaminated areas^8^. Thus, a threat to the sustainability of food production has been recognized as the other side of the As calamity^8^,^9^. Understanding the mechanism of As toxicity in plants is crucial to find a sustainable solution to the problem. Arsenic exists in various chemical forms in the environment, which differ considerably in plant uptake, mobility and toxicity. Thus, speciation of As in various plant parts is an important tool to understand the *in planta* transformation and metabolism of various As species.

Inorganic arsenate [HAsO_4_^2−^ or As^V^] and arsenite [H_2_AsO_3_^−^ or As^III^] are the predominant species in water and soil, however, in soil considerable amount of methylated arsenic species [methylarsonic acid; MA^V^ and dimethylarsinic acid; DMA^V^] may also be present due to microbial action^10^,^11^,^12^,^13^ or due to past uses of methylated As compounds (sodium salt of MA^V^ and DMA^V^ or cacodylic acid) as pesticides and herbicides^4^,^14^. Arsenate is taken up by the plants through phosphate transporters^15^, while As^III^ is taken up by nodulin26-like intrinsic (NIPs) aquaporin channels, along with neutral solutes like glycerol, ammonia and silicic acid^16^. The rice aquaporin Lsi1 also mediates the uptake of methylated arsenic species^14^. Arsenate, being a chemical analogue of phosphate (P), may replace P in phosphorylation reactions and interferes with the energy and P metabolism, while AsIII binds to the sulfhydryl groups of peptides and proteins hampering their activity^17^. The recent finding that at lower concentration, As predominantly accumulated in the nucleus of plant cell indicate that As may interfere with nucleic acid synthesis by replacing P^18^. Further, it has been observed that even a small amount of As reaching to the chloroplast may hinder pigment biosynthesis through inhibition of tetrapyrrole biosynthesis^18^,^19^. Whether exposed to As^V^ or As^III^, inside plant As^III^ predominates, demonstrating efficient reduction of As^V^ in the plant cells^20^,^21^,^22^,^23^. Arsenite is then either extruded from the roots^22^,^24^ or, due to high affinity for thiol groups, gets complexed with glutathione and/or phytochelatins (PCs) and stored within the vacuole, particularly in non-hyperaccumulator plants^23^,^25^,^26^,^27^. While in hyperaccumulators thiols have limited role and most of the As is stored as As^III^ 21,28. Trivalent methylarsonous acid (MA^III^) can also be complexed with PCs^23^,^25^. Rice (*Oryza sativa*) is particularly efficient in As accumulation, it accumulates 10 times more As than other cereal crops^4^,^29^. The anaerobic conditions in flooded paddy field enhance As bioavailability and may also favour As biomethylation^30^. Inorganic As (iAs; i.e. As^V^ + As^III^) and DMA^V^ are the main species present in rice grain and straw, though several other methylated As has also been detected in rice^10^,^12^,^13^,^31^,^32^. Rice contains hydroxymethyl-gluthathione (hm-GSH; having C-terminal Ser instead of Gly) in addition to glutathione (GSH) and induction of PCs and hydroxymethyl-PCs in response to Cd has earlier been reported in rice^33^. Recent studies have reported that PCs may have role in restricting iAs in rice roots^34^,^35^. Detoxification of MA^V^ in plants also involves increased production of non-protein thiols^36^. Nevertheless, presence of a significant amount of weakly bound As as well as free PCs in plants under As stress shows that not all synthesized PCs are used for As binding^23^,^25^. Since only trivalent As can be thiol complexed, reduction of pentavalent As may be a limiting step for thiol complexation. Therefore, direct determination of As-thiol complexes and various uncomplexed forms present in different plant parts is essential to reveal the role of vacuolar sequestration of complexes in As tolerance more conclusively. No comprehensive study has been performed to reveal transformation of inorganic and methylated As, extent of their complexation by thiols and nature of complexes in response to various As species in rice plant, and if the complexation of various As species in roots can be correlated with As translocation in shoot. Thus, in the present study, accumulation, translocation and speciation of inorganic (As^V^) and methylated As (MA^V^, DMA^V^) was investigated in roots and shoots of rice variety Triguna (*Oryza sativa* L., subsp. *indica*), a popularly grown variety of West Bengal, India to directly determine the role of thiol complexation in roots to restrict As translocation to shoots. The speciation through anion exchange chromatography coupled to ICP-MS was complemented with reversed-phase-HPLC simultaneously coupled to ICP-MS and ESI-MS to elucidate the diversity and extent of thiol complexation versus various weakly bound redox species of As in response to inorganic and methylated As.

## Results

Considering the *in planta* transformation of As species and redox state, hereafter iAs, MA and DMA were used for respective inorganic and methylated As species at the places where the redox states could not be explicitly specified.

### Accumulation and translocation of As^V^, MA^V^ and DMA^V^ and effect on plant growth

**Figure 1:**
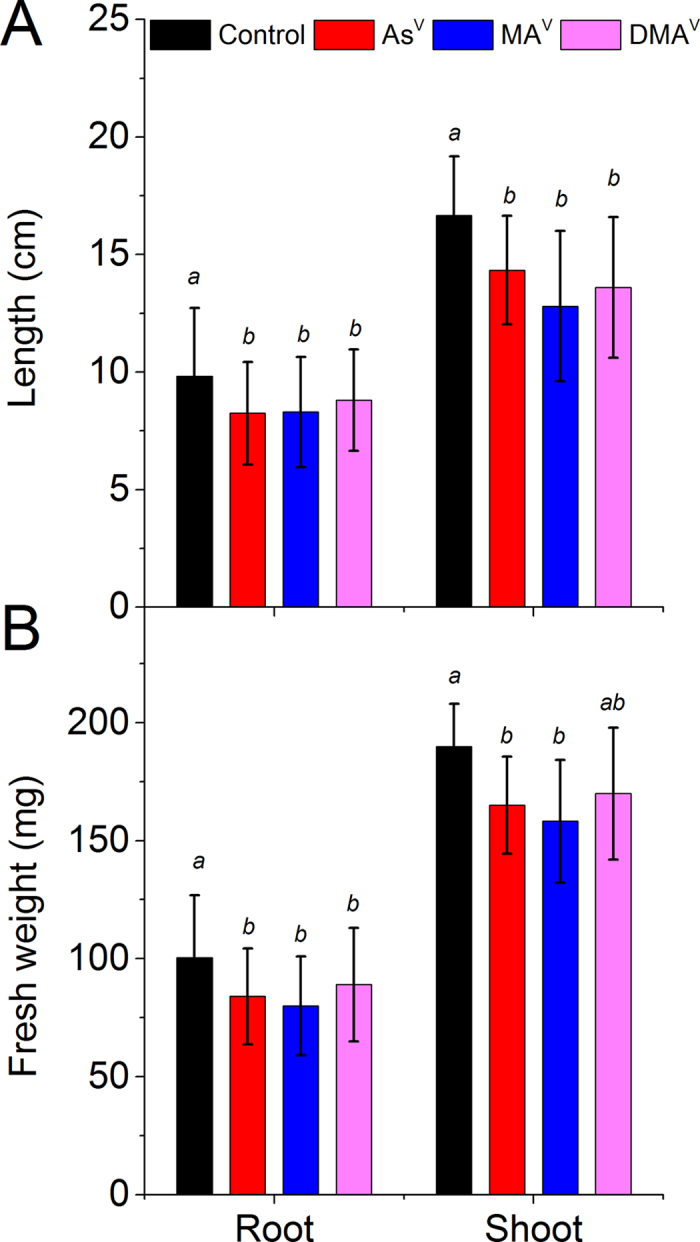
Growth of rice plant under control and after exposure to different species of As. Plants were exposed to10 μM As^V^ or 50 μM MA^V^ or 50 μM DMA^V^ for 7 d. (**A**) Root and shoot length, (**B**) Root and shoot fresh weight. Values are mean ± SD, n = 30. Different letters indicate significantly different values within root and shoot (P ≤ 0.05).

**Figure 2:**
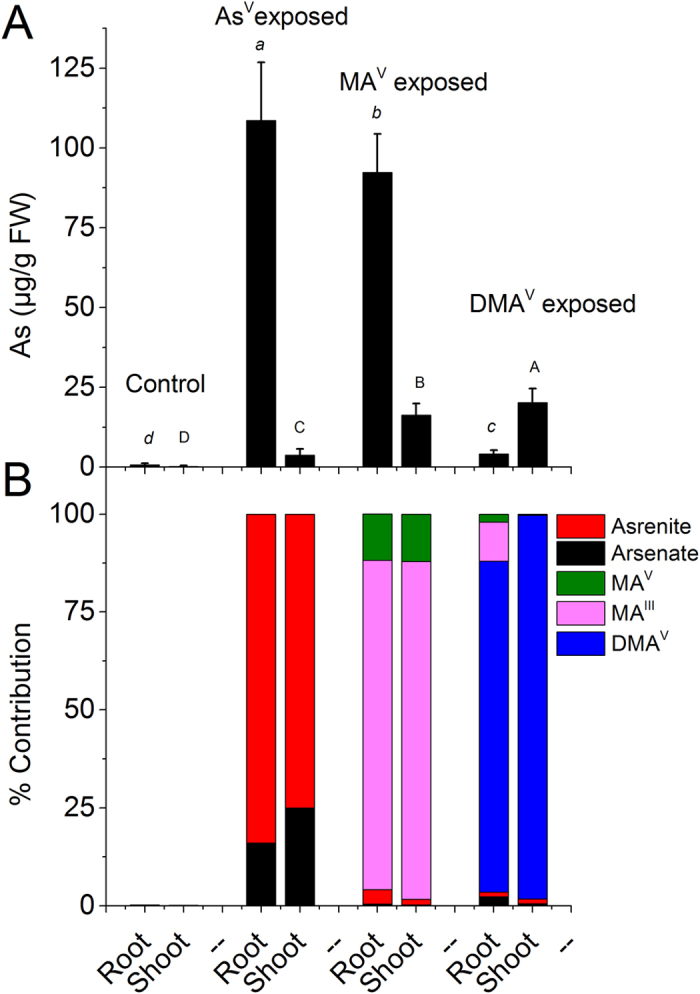
Total accumulation and speciation of As in root and shoot of rice plant under control and after exposure to different species of As. Plants were exposed to10 μM As^V^ or 50 μM MA^V^ or 50 μM DMA^V^ for 7 d, (**A**) Total accumulation of As (**B**) contribution of different As species separated through anion exchange chromatography coupled to ICP-MS. In control roots and shoots only traces of As^V^ and As^III^ were present. Values are mean ± SD, n = 12. Different letters indicate significantly different values between different arsenic species, small letters within roots and capital letters within shoots (P ≤ 0.05).

Moderately toxic concentrations of As^V^ (10 μM), MA^V^ and DMA^V^ (both 50 μM) were chosen for the investigation of accumulation and speciation of As on the basis of pre-experiment. The As^V^ at 20 μM caused severe toxicity after 7d (47 ± 8.3% inhibition in root length) (data not shown) while MA^V^ and DMA^V^ were moderately toxic until 50 μM. Further, the total accumulation of As (root + shoot) after 7 d was comparable in the plants exposed to 10 μM As^V^ and 50 μM MA^V^ (Supplementary Figure S3). Therefore, these concentrations were chosen for the further study to directly compare the root to shoot translocation and speciation of As. The concentration of DMA^V^ was taken similar to MA^V^. The growth of plant, in terms of root, shoot weight and length, was significantly reduced upon exposure to various As species in comparison to control (n = 30, P < 0.05). The maximum reduction in root and shoot weight was in MA^V^ exposed plants followed by As^V^ (Fig. 1). Maximum reduction in root length (16%) was in As^V^ exposed plants while in shoot length was (23%) in MA^V^ exposed plant. A significant accumulation of As was observed upon exposure to As^V^, MA^V^ or DMA^V^ (Fig. 2A). At equimolar exposure concentration (10 μM), the total amount of As accumulated by the plants (root + shoot) was approximately 3 and 14 times lower in MA^V^ and DMA^V^ exposed plants, respectively in comparison to As^V^ exposed plants (Supplementary Fig. 3). However, in As^V^ exposed plants most of the As was confined to the roots, only around 4% of the total accumulated As was transported to the shoots. While in MA^V^ and DMA^V^ exposed plants, around 15% and 81%, respectively, of the accumulated As was transported to the shoots (Fig. 2A). Thus, shoot/root transfer factor (TF) was 0.033 ± 0.012, 0.18 ± 0.05 and 4.91 ± 0.72 for As^V^, MA^V^ and DMA^V^, respectively.

### Speciation of As in rice exposed to As^V^, MA^V^ and DMA^V^ by means of anion exchange chromatography coupled to ICP-MS

**Figure 3:**
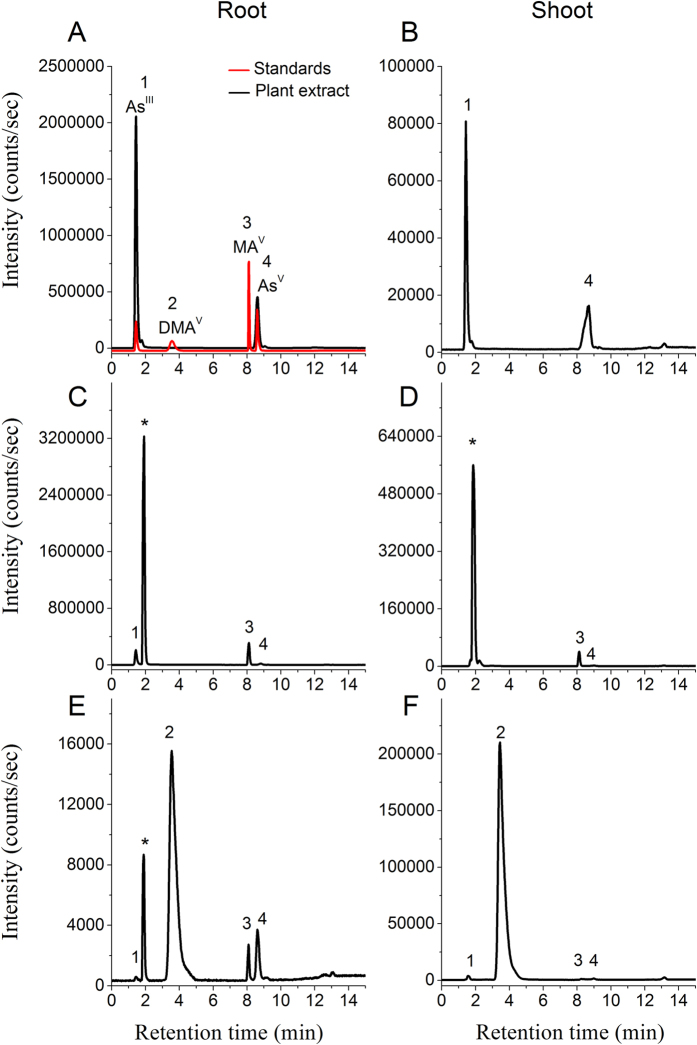
Speciation of As in fresh root and shoot extract of rice plant through HPLC (anion exchange IonPac^®^AS7 column) coupled to ICP-MS. Roots (left panel) and shoots (right panel) exposed to 10 μM As^V^ (**A**,**B**), 50 μM MA^V^ (**C**,**D**) and 50 μM DMA^V^ (**E**,**F**). In control roots and shoots only traces of As^V^ and As^III^ were present. Standards; (1) arsenite; As^III^, (2) dimethylarsinic acid; DMA^V^; (3) methylarsonic acid; MA^V^ and (4) arsenate; As^V^. Peak at 1.9 min shown with ‘*’ was identified as MA^III^ by synthesizing it through the method of Cullen *et al*.^53^ described in supplementary information.

Separation through anion exchange chromatography coupled to ICP-MS detector revealed As speciation (or transformation) in terms of oxidation state and presence of methylated and non-methylated species. The extraction efficiency of As was 91 ± 5.3% (n = 32), and the chromatographic recovery of As was 88 ± 7.9%. The ICP-MS chromatograms of fresh extracts of roots and shoots, exposed to As^V^, MA^V^ and DMA^V^, are given in Fig. 3. In the plants exposed to As^V^, only As^V^ and As^III^ were detected in both roots and shoots (Fig. 3A and B). Arsenite was the main species contributing up to 84% and 75% of the total As in roots and shoots, respectively (Fig. 2B).

Speciation analysis of MA^V^ exposed plants revealed that in both roots and shoots, most of the MA^V^ was reduced to MA^III^ (84 to 86% in roots and shoots, respectively) and about 12% was present as MA^V^. In roots a significant amount of As^III^ was also present and small quantities of As^V^ and As^III^ were detected in shoots as well (Figs 2B and 3C,D). Whereas, in DMA^V^ exposed plants most of the accumulated As remained as DMA^V^ (approx. 85% in roots and 98% in shoots). In the roots small amounts of As^V^, MA^III^ and MA^V^ were also detected, while in shoots only As^V^ and As^III^ were detected along with DMA^V^ (Figs 2B and 3E,F).

### Thiol complexation of As^V^, MA^V^ and DMA^V^ in rice by means of RP-HPLC-ICP-MS/ESI-MS

**Figure 4:**
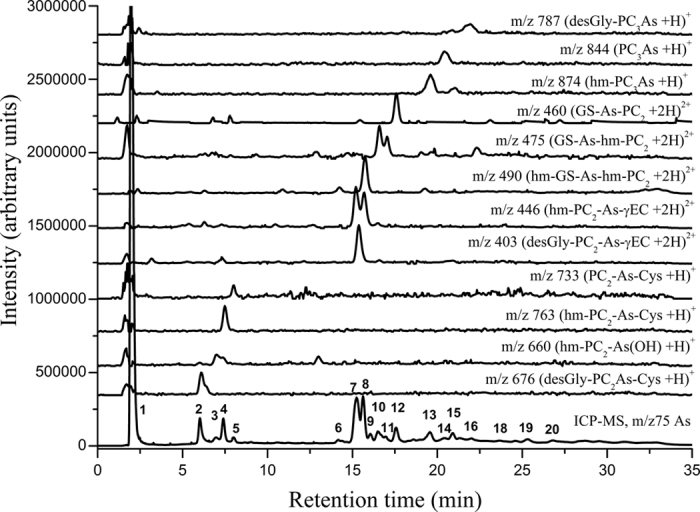
Chromatogram of As species in fresh root extract of rice plant exposed to 10 μM As^V^. HPLC was coupled to ICP-MS (*m/z* 75 As) and simultaneously to ESI-MS (*m/z* 676, 660, 763, 733, 403, 446, 490, 475, 460, 874, 844, 787). Peak 1 contains unbound As; peak 2 contains desGly-PC_2_As-Cys; peak 3 contains hm-PC_2_-As(OH); peak 4 contains hm-PC_2_-As-Cys; peak 5 contains PC_2_-As-Cys; peak 7 and 8 contains hm-PC_2_-As-γEC and desGly-PC_2_-As-γEC & hm-GS-As-hm-PC_2_ coeluted with peak 7 and 8 respectively; peak 10 and 11 contains GS-As-hm-PC_2_; peak 12 contains GS-As-PC_2_; peak 13 contained hm-PC_3_-As; peak 14 contained As-PC_3_; peak 15 contains desGly-PC_3_-As. All other peaks are yet unidentified As species.

**Figure 5:**
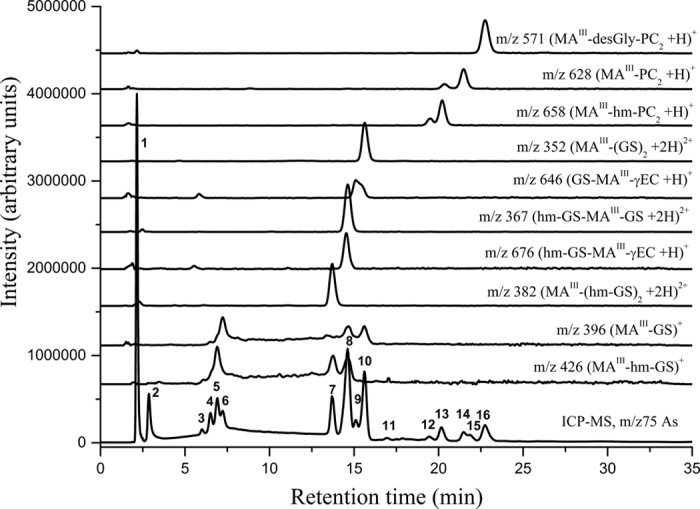
Chromatogram of As species in fresh root extract of rice plant exposed to 50 μM MA^V^. HPLC was coupled to ICP-MS (*m/z* 75 As) and simultaneously to ESI-MS (*m/z* 426, 396,382, 367, 676, 646, 352, 658, 628, 571). Peak 1 contains unbound As; peak 3 contains desGly-PC_2_-As-Cys; peak 5 contains hm-GS-As-CH_3_; peak 6 contains GS-As-CH_3_; peak 7 contains (hm-GS)_2_-As-CH_3_; peak 8 contains hm-GS-As(-CH_3_)-GS and hm-GS-As(-CH_3_)-γEC was coeluted; peak 9 contains GS-As(-CH_3_)-γEC; peak 10 contains (GS)_2_-As-CH_3_; peak 12 &13 contains hm-PC_2_-As-CH_3_; peak 14 contained PC_2_-As-CH_3_; peak 16 contained desGly-PC_2_-As-CH_3_. All other peaks are yet unidentified As species.

To see the level of thiol bound As and diversity among complexes, speciation through RP-HPLC coupled simultaneously to ICP-MS and ESI-MS was used. The molecular masses (mass-to-charge ratio; *m/z,* for [M + H]^+^ and [M + 2H]^2+^) and retention times of various As complexes and thiols have been given in Supplementary Table S1. Chromatographic separations of fresh extracts from As^V^ and MA^V^ exposed roots are shown in Figs 4 and 5 and of DMA^V^ exposed roots and shoots and MA^V^ exposed shoots are shown in Supplementary Fig. 4. The extraction efficiency of As was 78 ± 9.2% (n = 48), and the chromatographic recovery of As was 89 ± 14.5%.

A considerable diversity in As-thiol complexes involving Cys, GSH, γEC, hm-GSH, PCs, hm-PCs and des-Gly-PCs was observed. The hm-PC and des-Gly-PC variant of PCs were termed as respective homologues. The identification of complexes containing GSH, PC_2_, PC_3_ and des-Gly homologue of respective PCs was carried out by the comparison of the retention times of the As-selective chromatographic peaks (*m/z* 75, ICP-MS) with their characteristic retention times and characteristic *m/z* signals in ESI-MS as identified by the synthesized standard complexes given in Supplementary Table S1, while the complexes of hm-GSH and hm-PCs were characterized by characteristic *m/z* signals. The later were eluted slightly earlier than the corresponding As-PC complexes (Supplementary Table S1).

**Figure 6:**
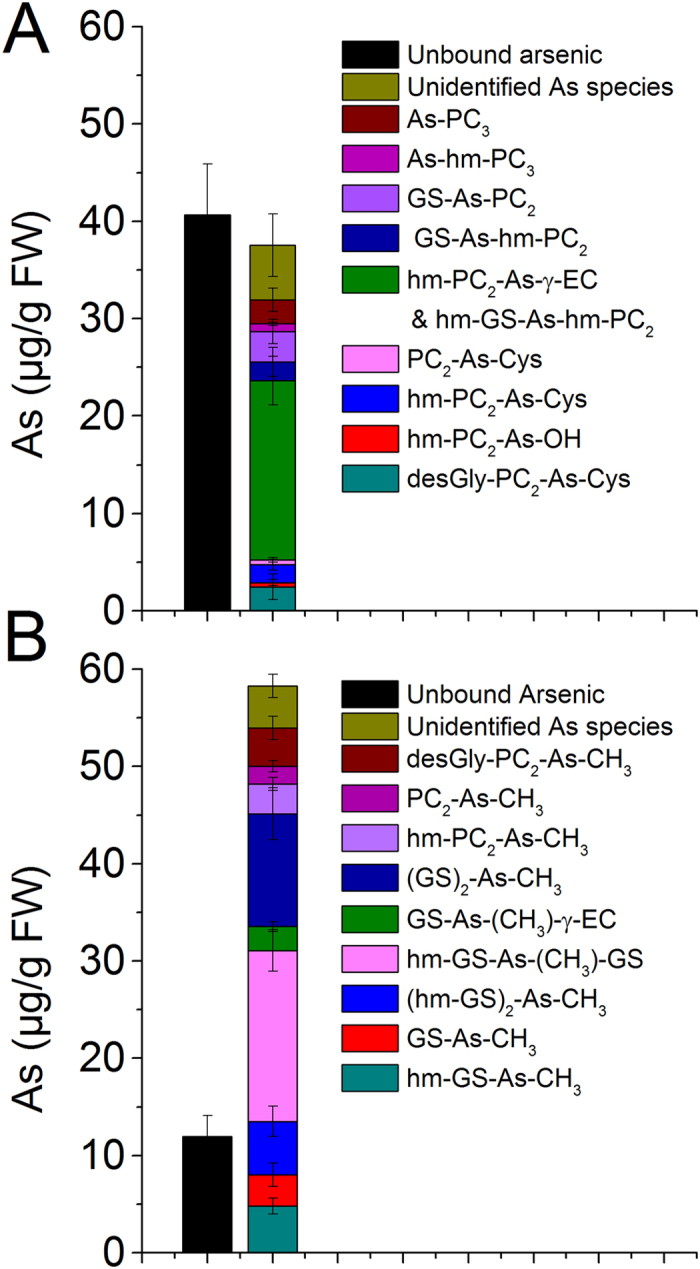
Quantitative determination of thiol complexed As species analyzed through HPLC-ICP-MS/ESI-MS in fresh root extract of rice plant exposed to As^V^ or MA^V^. (**A**) Level of different As species upon exposure to 10 μM As^V^, (**B**) Level of different As species upon exposure to 50 μM MA^V^. ICP-MS data (*m/z* 75) was used for the quantification. Values are mean ± SD, n = 12. FW, Fresh weight. In control root and shoot traces of unbound As was present.

The ICP-MS (*m/z* 75 As) data after chromatography in extracts of As^V^ exposed plant roots showed up to 20 As containing species (Fig. 4) binding up to 48 ± 6.4% of eluted As (Fig. 6A). Of which 12 were identified as complexes of PCs and homologous PCs. Nine out of 12 identified As species were mixed thiol complexes containing PC_2_ or its homologue with Cys, GSH, hm-GSH and γEC while other three complexes were As bound to PC_3_ and homologues of PC_3_ (Fig. 4). The peaks at 15.2 and 15.6 in the ICP-MS were the main As containing species binding up to half of the total bound As. This peak corresponded to hm-PC_2_-As-γEC (*m/z* 446) and hm-PC_2_-As-hm-GS (*m/z* 490; co-eluted at 15.6 min). All together PC_2_ and its homologue containing complexes contained about 76% while unidentified As species contained about 15% of complexed As. Only about 9% of the bound As was complexed with PC_3_ and hm-PC_3_ (Fig. 6A). In shoots of As^V^ exposed plants, most of the As was eluted unbound. Only 2–6% of the total shoot As was thiol complexed mostly in the form of hm-PCs viz. hm-PC_2_-As-γEC, hm-PC_2_-As-hm-GS; hm-PC_2_-As-GS and hm-PC_3_ (Supplementary Fig. 5).

In MA^V^ exposed rice roots up to 16 As containing species were observed, of which 11 were identified as thiol bound (Fig. 5). Peak 1 is unbound As containing approximately 17 ± 4.3% of the eluted As. While a large proportion of As was eluted as various thiol bound trivalent methylarsonous acid (MA^III^). ICP-MS chromatogram showed that the MA-thiol complexes were eluted in three well separated groups, first group contained MA^III^ bound to one molecule of GSH or homologues; second group contained MA^III^ bound to two molecules of GSH or homologues as various mixed complexes; while in third group MA^III^ was bound to PC_2_ or homologues. The peaks at 2.9, 6.5, 16.9 and 21.9 min (no. 2, 4, 11 and 15, respectively) are yet unidentified As species. Approximately 78% of the total bound As in MA^V^ exposed plant was bound to GSH and homologues of GSH with hm-GS-As-(CH_3_)-GS and (GS)_2_-As-CH_3_ as main species, while 16% was bound to PC_2_ and homologues of PC_2_. No PC_3_ or hm-PC_3_ containing complex was identified (Fig. 6B). In shoots only uncomplexed As and an unidentified species at 2.9 min (containing 7.8 ± 2.6% of total shoot As) was observed (Supplementary Fig. S4).

In DMA^V^ exposed roots and shoots of plant most of the As was eluted as DMA^V^. In shoot, an unidentified As species at 3.9 min in ICP-MS data was observed, which contained 2.3 ± 1.1% of shoot As (Supplementary Fig. S4).

### Level of free thiols

**Figure 7:**
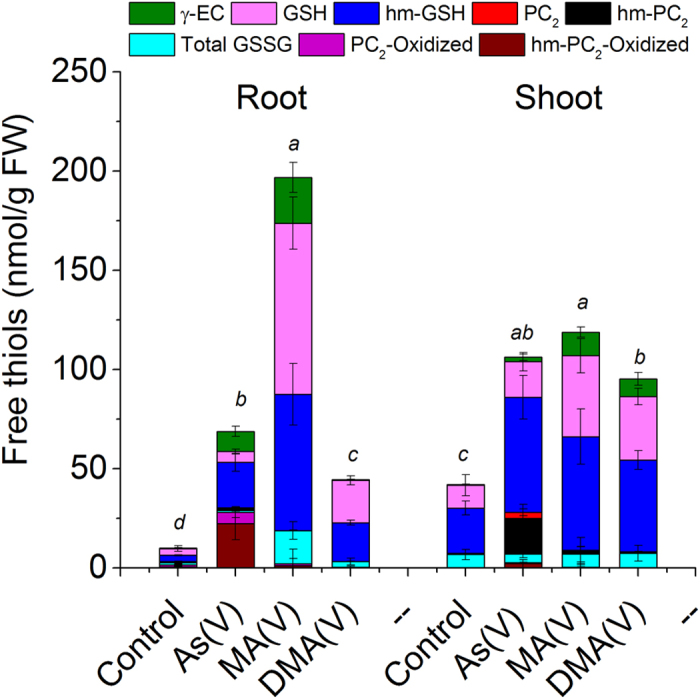
Level of free thiols in roots and shoots of rice plant under control and after exposure to different species of As. Plants were exposed to10 μM As^V^ or 50 μM MA^V^ or 50 μM DMA^V^ for 7 d. ESI-MS data in single ion monitoring (SIM) mode was used for quantification of thiols. Total GSSG includes GSSG, hm-GSSG and hm-GSSG-hm. Values are mean ± SD, n = 12. FW, Fresh weight. Different letters indicate significant difference between exposures to different arsenic species in roots and shoots (P ≤ 0.05).

In control plants, shoots contained higher level of thiols than roots. A large amount of free oxidized and reduced thiols were detected in As exposed plants as well (Fig. 7). The separation of various thiols and their identification by ESI-MS have been shown in Supplementary Fig. S6. Both in roots and shoots of control plants, GSH and hm-GSH were the main free thiols, small amounts of oxidized species were also present. Exposure to As^V^, MA^V^ or DMA^V^ significantly increased the level of free thiols (n = 30, P = < 0.001). The maximum increase in free reduced thiols (mainly GSH and hm-GSH) was observed in MA^V^ exposed plants with 24 and 3 fold increase in roots and shoots, respectively, in comparison to control. While in As^V^ exposed plants the level of free reduced thiols was approx. 5 and 2.8 fold higher and in DMA^V^ exposed plants approx. 5.5 and 2.8 fold higher than the roots and shoots of control plants, respectively. Arsenate exposure predominantly increased the level of hm-GSH over GSH, whereas in MA^V^ and DMA^V^ exposed plants both hm-GSH and GSH were increased. Also in As^V^ exposed plants, significant increase in free PC_2_ (mostly hm-PC_2;_ P < 0.001) was observed in shoots while roots mostly contained oxidized PCs (P < 0.001). Further, oxidized thiols only increased in the roots of As^V^ and MA^V^ exposed plants while no significant difference was observed in shoots in comparison to respective controls. A significant increase in the level of γEC was also observed in roots and shoots upon As^V^ and MA^V^ exposure (P = < 0.001). Traces of free PCs were present in shoots of MA^V^ and DMA^V^ exposed plants, however, no free PC_3_ or its homologue was observed in any treatment.

## Discussion

Rice is the major dietary exposure route of As, particularly of inorganic As (iAs). In India, West Bengal is highest rice producing state and unfortunately it is most severely As contaminated state as well. In the recent years yield loss due As toxicity has been recognized as a serious problem affecting the sustainability of food production in South-East Asian countries^8^,^9^. Triguna, a high yielding rice variety of West Bengal, India was chosen for the present study as it is more tolerant to As among other popular varieties^37^. The accumulation, transformation and thiol complexation of various As species, commonly present in soil and water, was analyzed to unravel mechanism of As metabolism in rice.

The level of iAs supplied was well below the levels found in ground water as well as in soil solutions of contaminated areas^8^,^9^. Though the level of methylated As is generally low in soil, however, depending on soil conditions and historical use of arsenicals as pesticides and herbicides, use of poultry litter and other organic fertilizer may result in high level of organic As in soil^4^,^13^. Organic manure application has been reported to cause increased methylation of As in soil as well as more release of arsenic into the soil solution^13^.

In the present study, the growth of the selected rice variety was significantly reduced under As stress. However, the reduction in growth was not significantly different among the various treatments (e.g. As^V^, MA^V^ and DMA^V^). Roots are the first site of As exposure from soil and water and the initial source of arsenic to shoots and grains. Thus, As metabolism in roots is crucial for determining its transport and toxicity to shoots and effect on yield. While hyperaccumulators transport most of the As to shoots^21^,^38^, retention of As in roots is a general phenomenon observed in non-hyperaccumulator plants to avoid shoot toxicity^39^,^40^,^41^. In the present study the root uptake (total As in root and shoot) and accumulation in root was maximum for iAs followed by MA and DMA while the accumulation in shoot was in revers order. Higher root to shoot mobility of methylated As in comparison to iAs has been found in plants including rice in earlier studies as well^14^,^42^,^43^,^44^. Sequestration of thiol complexed As in the root vacuoles has been suggested for the retention of As in roots. In the present study, most of the As^V^ and MA^V^ were reduced to As^III^ and MA^III^ in roots which is essential for complexation with thiols, while DMA^V^ was largely untransformed. Thus, explaining the higher mobility of DMA. In shoots of As^V^ exposed plants, the proportion of As^V^ was higher than in root. Arsenite has been reported to be the dominant species transported to the shoot in rice, though small amount of As^V^ may also be transported through P transporters^7^. Thus, it may be possible that As^III^ in the shoots was primarily transported from the roots. In contrast, MA^V^ exposed plant shoots exhibited a large proportion of the accumulated As as MA^III^ (86%) which is significantly higher than in previous reports. Lomax *et al*.^35^,^45^ reported predominance of MA^III^ (about 65%) in roots but only small amounts of MA^III^ (8.6%) in shoots of rice. In contrast, Li *et al*.^14^ found only up to 16% MA^III^ in rice roots, while in shoots no MA^III^ was present. The short exposure duration of 1d could be an explanation for low reduction of MA^V^ in the later study. Probably the reduction of MA^V^ is not as rapid as observed for As^V^ in rice roots^22^. Further, MA^III^ is highly unstable and may rapidly oxidize to MA^V^ during extraction, therefore the complete anoxic condition during extraction till analysis could be another factor for high MA^III^ detection in the present study. The small amount of As^III^ and As^V^ detected in the roots and shoots of MA^V^ and DMA^V^ exposed plants may derive from the stock solutions. The stock solution of MA^V^ contained 5% iAs and traces of iAs was also detected in DMA^V^ stock solution. However, presence of MA^V^ and MA^III^ in DMA^V^ exposed plant cannot be explained. Probably these derived from the demethylation in the rice roots as suggested by Lomax *et al*.^45^.

The higher retention of As in roots of As^V^ exposed plants in comparison to MA^V^, however, did not correlate well with the proportion of their reduction, thus possible complexation and vacuolar sequestration. Therefore, direct determination of thiol complexation was important to explore the extent of thiol complexation in the plants exposed to various As species. Speciation through RP-HPLC coupled to ICP-MS and ESI-MS in parallel has shown to be reliable technique for determination of diversity of ligands and As-thiol complexes in plants, as well as with respect to the stability of complexes^25^,^46^,^47^. Artifacts during sample preparation, such as complex dissociation or ligand exchange may occur, but could be diminished by low temperature and inert gas treatment up to measurement. However, the results of chromatographic speciation in fresh plant extract and *in situ* speciation through XAS technique in intact plant tissue were in good agreement in aquatic plant *Ceratophyllum demersum*^23^. In the present study as well almost no change in the level of various As species was observed during the analysis period. Therefore, this technique was used for the direct determination of As-thiol complexes, their quantification and diversity in rice under inorganic and methylated As exposure. Rice and many other species of family Poaceae contain hm-GSH along with GSH. Cadmium induced synthesis of hm-PCs in rice have been reported by Klapheck *et al*.^33^. Present study demonstrated that both As^V^ and MA^V^ exposure induced the synthesis of hm-PCs in addition to regular PCs in rice and a considerable diversity in As-thiol complexes exist. The complexes of As^III^ and MA^III^ with hm-GSH and hm-PCs as well as other mixed complexes involving γEC and des-Gly-PCs have been identified for the first time in the present study. In As^V^ exposed plants about 48% As was eluted as various thiol complexes. Most of the complexes were of PC_2_ types (i.e. PC_2_, hm-PC_2_ and des-Gly-PC_2_) with variable third ligand viz. water, Cys, γEC, GSH or hm-GSH binding up to 70–80% of the total As-thiol. The maximum As was complexed with hm-PC_2_ containing complexes (approximately 60% of the total As-thiol). While PC_3_ and hm-PC_3_ contributed only about 9% of the As-thiol. In the present study, neither As-(GS)_3_ nor As-(PC_2_)_2_ or their homologue complexes were identified. *In vitro* complexation showed that GSH and Cys are less preferred ligands over PCs^23^. In sunflower (*Helianthus annuus*) As-PC_3_ was the preferred complex^25^, and in aquatic plant *Ceratophyllum demersum* As-(PC_2_)_2_ was the main complex^23^. In the present study only small amounts of free reduced PC_2_ and hm-PC_2_ were present in roots while free PC_3_ or homologues were not detected at all. These indicate that PCs (particularly PC_3_) are most preferred ligand, if available, due to more stability of the complexes, however, their synthesis might be limiting. Further, accumulation of large amount of upstream metabolites of the PC biosynthetic pathway e.g. γEC, GSH and hm-GSH in roots also indicates that phytochelatin synthase (PCS) activity was the limiting step in synthesis of PCs. Therefore, mixed GS-As-PC_2_ type complexes were preferably formed. In shoots only a small fraction (2–6%) of the total shoot As was in the form of As-thiol complex. Since whole areal part (2mm above the root-shoot Junction) was used for the speciation analysis, it could be possible that most of the shoot As was contributed from the vascular tissues (xylem and phloem) where it remained uncomplexed^20^,^43^. In mesophyll also most of the As may remain uncomplexed^23^. Probably shoot As tolerance does not rely on thiol complexation in shoots but on reduced As translocation by complexing As in roots and by suppressing P transporters as reported by Begum *et al*.^35^.

In MA^V^ exposed rice roots about 83% of the eluted As was thiol complexed. This is the maximum level of complexation observed in the plant extract. Since speciation through anion exchange chromatography revealed presence of approximately 84% MA^III^ in the roots of MA^V^ exposed plants, therefore it seems that most of the MA^III^ in root was complexed with thiols. In contrast to As^III^ complexes, for MA^III^, GSH and hm-GSH were the preferred ligands binding up to 78% of total bound MA^III^. Probably methylated As is not a strong inducer of PCS as suggested by Raab *et al*.^25^. However, in contrast to their hypothesis, present study showed high MA^III^-thiol complex formation. It seems that MA^V^ was efficiently being reduced to MA^III^ which has high affinity for thiol group, thus, immediately binding to available thiols e.g. GSH and hm-GSH, and could induce GSH biosynthesis probably in a demand driven manner. Nevertheless, high MA-thiol complexation and diversity of complexes observed in rice seems a plant species specific response. Since in *Ceratophyllum demersum*, exposed to MA^V^ for same concentration and duration, only MA-PC_2_ was detected binding up to 35% of As (unpublished data). In shoots, despite the predominance of MA^III^, only little was thiol-complexed.

In DMA^V^ exposed plants, though induced synthesis of thiols was observed in roots and shoots, no DMA-thiol complex could be identified. Only an unidentified species accounting for about 2.3% of total shoot As was observed.

**Figure 8:**
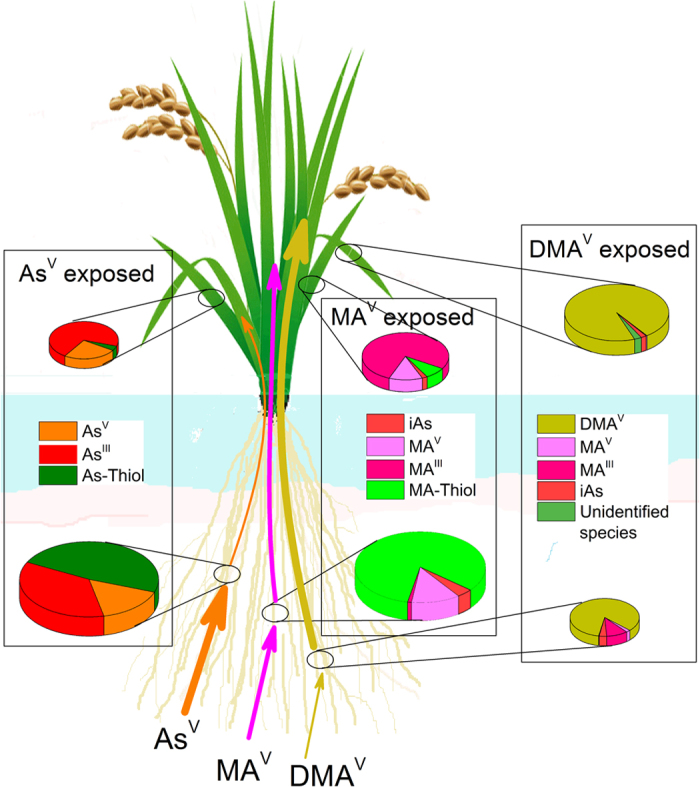
Various species of As present in the root and shoot of rice plant exposed to As^V^, MA^V^ or DMA^V^. The overall speciation was derived by combining the results of anion exchange chromatography coupled to ICP-MS which delivered redox states, and RP-HPLC-(ICP-MS)-(ESI-MS) which delivered unbound *vs.* thiol-bound As and considering that only As^III^ and MA^III^ can be complexed with thiols. The thickness and the colour of arrows shows swiftness of uptake and translocation of As by rice exposed to respective As species and the size of Pie disc shows relative As content in root and shoot.

From the above it is clear that despite more complexation of MA in roots a higher proportion was translocated to shoots than iAs. By combining the results of both speciation methods [redox state observe through anion exchange chromatography coupled to ICP-MS and unbound *vs.* thiol-bound observed through RP-HPLC-(ICP-MS)-(ESI-MS)] in a complementary manner and considering that only As^III^ and MA^III^ can be complexed with thiols, the extent of unbound As^III^ and MA^III^ along with MA^V^, As^V^, As-thiol and MA-thiol can be elucidated (Fig. 8). This shows that in MA^V^ exposed plants only little MA^III^ (about 1%) was uncomplexed in roots while in As^V^ exposed plants a large proportion of As in roots was present as weakly bound As^III^ (Fig. 8). These results indicates that the thiol-complexation in roots might not be the limiting factor for shoot transport of MA, but the reduction of MA^V^ in roots could be one of the limiting factor. It could be possible that the reduction of MA^V^ was slow during the initial phase of exposure, thus resulting in significant MA^V^ transport to the shoots before complexation in the roots. Ye *et al*.^43^ found only MA^V^ in xylem exudate of *Ricinus communis*. However, it is still inconclusive until the rate of MA^V^ reduction with respect to time is studied. The lower transport of iAs despite rapid reduction to As^III^, a preferred species for shoot translocation in rice^7^ may be explained by the efflux of As^III^ by rice roots in addition to thiol complexation^22^. Recently a novel arsenate reductase HAC1 has been shown to play important role in iAs tolerance by reducing As^V^ in the outer cell layer of the roots, facilitating efflux of As^III^ out of the roots thus limiting its transport to shoot^48^. HAC1 is also present in the pericycle and its likely role in limiting the arsenic loading into the xylem has been suggested. It is possible that in rice As^V^ influx was in equilibrium with As^III^ efflux and thiol complexation after reduction, thus only little As was loaded to the xylem for shoot transport. The rice aquaporin Lsi1 has been shown to play a role in arsenite efflux in rice roots^24^. Thus it seems that a synchronized involvement of thiol-complexation and vacuolar sequestration in roots and efflux out of root cells are important to restrict iAs translocation to shoots.

The presence of large amount of free oxidized and reduced thiols in the roots and shoots upon exposure to both inorganic and methylated As, including DMA, points towards their other roles, such as, in maintaining the cellular redox equilibrium under As stress. PCs might also act as reducing agent similar to GSH possibly against As induced oxidative stress as evident by the presence of large amount of oxidized PC_2_ and hm-PC_2_ in roots. Tang *et al*.^36^ reported that thiols did not play a role in detoxification of DMA. In the present study as well no thiol complexation with DMA was observed, however increased synthesis of thiols may suggest their role as antioxidants against oxidative stress caused by DMA^36^. The large amount of thiols present in shoots of As^V^ and MA^V^ exposed plants points their increased synthesis probably to meet the high demand in roots and they are being transported to roots. The *cad2-1* mutant of Arabidopsis showed efficient transport of γEC, GSH and PCs from shoots to roots upon expression of bacterial γ-glutamylcysteine synthetase gene in shoots and showed an enhanced As^V^ tolerance over wild type^49^.

Both inorganic and methylated As has been shown to be toxic to the plants. Inorganic As inhibited pigment biosynthesis and growth and accumulated preferably in nucleus at low concentrations^18^,^19^ showing that even the small amount of iAs translocated to shoots can effect growth and yield of plant^8^. DMA, due its higher *in planta* mobility, can be more toxic than iAs, particularly for reproductive tissues^36^,^50^. Further, the strong affinity of MA^III^ for thiols, found in the present study, indicate its high reactivity which could make it potentially toxic for plants and animals. Exposure to MA has been reported to cause various toxicity symptoms including straighthead in rice, a physiological disorder of rice characterized by sterility of the florets leading to reduced grain yield^51^,^52^.

## Conclusion

The results of the present study showed that reduction and thiol complexation in roots are important steps for As^V^ and MA^V^ metabolism in rice but not for DMA^V^. Arsenate and MA^V^ both induced the synthesis of thiols in rice and a diversity of As^III^- and MA^III^-thiol complexes were formed in rice roots including various homologues of PCs and GSH. Thiol complexation in roots may restrict As translocation to shoots, however, despite higher complexation a slower reduction of MA^V^ seems to be limiting for MA sequestration in roots when compared to As^V^. Though, a time course analysis of MA^V^ reduction and MA^III^ complexation would give more conclusive results. Nevertheless, the predominance of MA^III^ in roots and shoots observed in the present study showed that during the growth period the rice plant may accumulate high level of MA^III^ in shoots which may also be transported to the grains. Thus, plants grown in the soils having high methylated As species, either due to pesticide treatment or high methylation rate of inorganic As, may not only contain high level of As in shoots and edible parts of the plants but also a potentially more toxic form of As for humans and animals.

## Materials and Methods

### Plant material and cultivation

The seeds of Rice (*Oryza sativa* L., subsp. *Indica, cv*. Triguna) purchased from authenticated seed supplier of Kolkata, West Bengal were grown in hydroponics. Seeds were surface sterilized with 0.5% NaOCl for 15 min, rinsed thoroughly with deionized water and soaked overnight. The seeds were then transferred to Petri-dishes containing moist filter papers and incubated at 28 °C in the dark for germination. After 5 d the uniform seedlings were selected and transferred to ‘Araponics’ hydroponic growing system (Araponics SA, Liège, Belgium, one seedling/seed holder; eighteen plants per tray). This consists of a black plastic tray with 2 L resident volume covered by a black lid with seed holders which supports the growth of 18 plants. The trays were completely filled with the nutrient solution (2 L) so that the roots were immersed in the solution. The composition of the nutrient solution was as follows: 2 mM Ca(NO_3_)_2_, 1 mM MgSO_4_ × 7H_2_O, 0.5 mM K_2_HPO_4_, 0.1 mM KCL, 10 μM H_3_BO_3_, 0.25 μM MnSO_4_ × H_2_O. 0.2 μM Na_2_MoO_4_ × 2H_2_O, 0.5 μM NiSO_4_ × 6H_2_O, 0.5 μM CuSO_4_ × 5H_2_O, 0.5 μM ZnSO_4_, 50 μM Fe-EDTA and 50 μM FeCl_3_ × 6H_2_O. The pH was adjusted to 5.8 by using 0.1 M KOH or HCl and the half of the nutrient solution was replaced with fresh solution every day. After 10 d of growth in controlled environment (14 h light period 250–300 μEm^−2^s^−1^, temperature 28–20 °C day/night), the plants were exposed to As^V^ (10 μM), MA^V^ or DMA^V^ (50 μM each) [as Na_2_HAsO_4_.7H_2_O (Alfa Aesar), (CH_3_)HAsNaO_3_ (Luxembourg Industries (PAMOL) Ltd. Tel-Aviv (Israel)) and (CH_3_)_2_NaAsO_2._3H_2_O (Sigma), respectively] and the level of K_2_HPO_4_ and KCl was changed to 10 μM and 1 mM respectively. After 7 d of exposure to various As species the plants were harvested and used for the analysis of various parameters (Supplementary Fig. S1).

### Harvesting of plants and growth measurements

After harvesting, the roots were rinsed by immersing in ice cold 2 mM phosphate buffer (pH 6.0) for 15 min followed by twice in ice-cold deionized water for 2 min. The plants were shaken to remove surface water and the root and shoot length of the plants were measured. The roots and shoots were then separated, weighed and immediately frozen in liquid nitrogen.

### Analyses of total As

Total As was measured in the fresh plant material after acid digestion (HNO_3_-H_2_O_2_) in a microwave digester^23^. A sector field inductively coupled plasma mass spectrometer (ICP-MS; Element XR, Thermo Fisher Scientific, Waltham, MA, USA) was used for the analysis. Prior to analysis, ICP-MS parameters were optimized every day and the calibration was verified using the following reference materials: SLRS-5 (River Water Reference Material for Trace Metals, NRCC), SPSSW1 (Surface Water Level 1, Spectra Pure Standards) and SRM 1643e (Trace Elements in Water, NIST). An addition of rhodium (4 μg L^−1^) to all samples was used for internal standardization.

### Sample preparation for As speciation and As-thiol complex analyses

The root and shoot were immediately frozen in liquid nitrogen, ground and aliquoted (200–400 mg) in pre-cooled and weighed microfuge tubes. The samples were immediately extracted for As speciation and thiol complexation as described earlier^23^. Briefly, an aliquot was immediately extracted in 1% degassed formic acid (90 min at 1 °C), centrifuged for 5 min at 1 °C and extract was quickly transferred to sample vial on ice and kept at 4 °C in the HPLC autosampler for the determination of As-thiol complexes. Another aliquot was extracted in degassed deionized water for 18 h on a tube rotator at room temperature. The extract, after centrifugation, was used for As speciation through anion exchange chromatography. All the handling, before and after the extraction, was done under nitrogen environment in a glove box. Total As concentrations in the extracts were also determined. Other subsamples were digested for the determination of total accumulation by the plant and for the determination of extraction efficiency by ICP-MS as mentioned above.

### Instrumental setup for arsenic speciation analysis

The separation and identification of the As species present in fresh plant extracts was performed using HPLC-ICP-MS. An IonPac^®^AS7 anion exchange column (Dionex, Sunnyvale, USA) was used for the separation of As species on a 1100 Series HPLC system (Agilent Technologies) coupled to VG PQ EXCELL ICP/MS (Thermo Elemental). A gradient of 0.04 mM HNO_3_ and 50 mM HNO_3,_ (0–5 min, 0%; 5–5.2 min, 0–10%; 5.2–11 min, 10%; 11–11.2 min, 10–100%; 11.2–13 min, 100%; 13–13.2, 100–0.0%; 13.2–18 min, 0.0% of 50 mM HNO_3_) was used to elute various inorganic and methylated As species. NIST 1568a rice flour was analyzed for analytical quality Control (total sum of species 279 ± 3 μg kg^−1^ with 32% iAs and 64% methylated As). Standards of arsenite (sodium arsenite solution, 0.05 M, Merck, Darmstadt, Germany), arsenate (Arsenic standard 1000 mg As L^−1^ [As_2_O_5_ in water] Titrisol, Merck), dimethylarsinic acid (cacodylic acid, Alfa Aesar, Karlsruhe, Germany) and methylarsonic acid (Luxembourg Industries (PAMOL) Ltd. Tel-Aviv (Israel)) were used for the identification of As species in plant extracts. For identification of MA^III^, it was synthesized by the method of Cullen *et al*.^53^ and identified through HPLC-(ICP-MS)/(ESI-Q-TOF-MS) as described in the supplementary information (Supplementary method & Supplementary Fig. S2).

### Instrumental setup for arsenic-thiol complex analysis

For the separation and identification of the As-thiol species present in fresh plant extracts, HPLC coupled online to ESI-MS (molecule-specific detector) and ICP-MS (element-specific detector) in parallel was used. The HPLC and detector conditions were used as described earlier^23^. Briefly, an Atlantis reversed-phase dC18 analytical column (4.6 × 150 mm, 5 μm particle size; Waters) was used for the separation of As-PC complexes on an 1100 Series HPLC system (Agilent Technologies). A gradient of 0.1% (v/v) formic acid (A) and 0.1% formic acid in 20% (v/v) methanol (B) was used (0–20 min, 0%–75% B; 20–30 min, 75% methanol; 30–30.10 min, 20%–0% B; 30.10–35 min, 0% B). Postcolumn, the flow was splitted with a ratio of 1:1 into the ICP-MS and ESI-MS devices. ESI-MS (6130 quadrupole LC/MSD; Agilent Technologies) was used in the positive ionization mode from *m/z* 120 to 1200 or in the single ion monitoring mode with the electrospray ionization source. For the identification of As species, the characteristic retention time and *m/z* in ESI-MS along with trace of As (*m/z* 75) at the corresponding retention in ICP-MS (7500ce; Agilent Technologies) was applied. For the quantification of As species, only ICP-MS data (*m/z* 75) were used. Dimethylarsinic acid was used as a standard for the calibration and quantification of all the As species. The interfering influence of chloride (ArCl^+^) and calcium (CaCl^+^) was proofed. First plant sample was repeated at the end of each batch to observe the changes during analysis period (typically less than 5 h for each batch).

### Synthesis of standards

Standards of PC_2_, PC_3_, PC_4_, desGly-PC_2_ and desGly-PC_3_ (PC without the C-terminal Gly) of 90–95% purity (in 6.3 mM diethylenetriaminepentaacetic acid/0.1% trifluoroacetic acid) procured from Clonestar Peptide Services were used for synthesizing various standards of As-thiol complexes and applied to HPLC-ESI-MS/ICP-MS. For synthesizing the complexes, reaction mixtures [5 mM As^III^/ MA^V^, 200 μM each PC and/or 2 mM GSH, and 10 mM Cys] were prepared in 0.1% degassed formic acid under nitrogen and kept overnight under nitrogen. The retention times and characteristic *m/z* values of standards in ESI-MS along with traces of As (*m/z* 75) in ICP-MS were used for the identification of complexes in plant extracts. The complexes involving hm-GSH and hm-PCs were identified by their characteristic *m/z* signal and the retention times relative to respective GSH and PC complexes (eluted slightly earlier than GSH and respective PC complexes as shown in Supplementary Table S1).

### Measurement of free thiols

For the determination of free reduced and oxidized thiols HPLC-ESI-MS data was used. PCs and desGly-PCs were identified by characteristic *m/z* signals and retention times using the standards, while hm-GSH and hm-PCs were eluted slightly earlier (Supplementary Fig. S2). For quantification of thiols ESI-MS data in single ion monitoring (SIM) mode was used. The calibration curve of reduced glutathione (GSH), PC_2_, PC_3_ and PC_4_ was used for quantification of respective thiols and homologues e.g. γ-glutamylcysteine (γEC), hm-GSH, desGly-PCs and hm-PCs while oxidized glutathione (GSSG) was used for the quantification of all the oxidized species.

### Statistics

Analysis of variance (ANOVA) was done using SigmaPlot 11 (SPSS Science, USA) at a significance level of P < 0.05. For significant effects, a post-hoc all-pair wise comparison via Student-Newman-Keuls Method in one way ANOVA, and via Holm-Sidak method for two-way ANOVA was performed.

## Additional Information

**How to cite this article**: Mishra, S. *et al*. Accumulation and transformation of inorganic and organic arsenic in rice and role of thiol-complexation to restrict their translocation to shoot. *Sci. Rep.* **7**, 40522; doi: 10.1038/srep40522 (2017).

**Publisher's note:** Springer Nature remains neutral with regard to jurisdictional claims in published maps and institutional affiliations.

## Supplementary Material

Supplementary Information
