# Supplementary material for: Accumulation and transformation of inorganic and organic arsenic in rice and role of thiol-complexation to restrict their translocation to shoot
Source: Sci Rep. 2017 Jan 17;7:40522. doi: 10.1038/srep40522 (PMC5240135; doi:10.1038/srep40522)
Supplement: Supplementary Information [file srep40522-s1.pdf]

Supplementary Information:

Accumulation and transformation of inorganic and organic arsenic in rice and role of thiol-complexation to restrict their translocation to shoot

Seema Mishra[1,2]*, Jürgen Mattusch[1] and Rainer Wennrich[1]

[1]UFZ – Helmholtz Centre for Environmental Research, Department of Analytical Chemistry, Permoserstr. 15, D-04318 Leipzig, Germany

[2]CSIR-National Botanical Research Institute, Plant Ecology & Environmental Science Division, Rana Pratap Marg, Lucknow 226 001 (U.P.), India

*Corresponding author:

Dr. Seema Mishra

CSIR-National Botanical Research Institute, Plant Ecology & Environmental Science Division, Rana Pratap Marg, Lucknow 226 001 (U.P.), India

Ph: +91-522-2297825; Fax: +91-522-2205836

E-mail: seema_mishra2003@yahoo.co.in

Supplementary Table S1. GSH, PCs and their arsenic complexes, molecular masses $[M+H]^+/[M+2H]^{2+}$, and retention time

Chemical Species	*m/z*	Retention times (min)
γ-EC	251	3.55
GSH*	308	3.58
hm-GSH	338	3.40
GS-SG*	613/307	6.63
hm-GS-SG	643/322	6.04
hm-GS-SG-hm	673/337	5.61
desGly-PC_2*	482	7.93
PC_2*	540	10.1
hm-PC_2	570	9.70
PC_2(ox.) *	538	11.97
hm-PC_2 (ox.)	568	11.63
PC_3*	772	17.22
PC_3 (ox.) *	770	19.12
Complexes identified in As^{V**} exposed plants		
Cys-As-desGly-PC_2*	676/338.5	6.02
Cys-As-$(GS)_2$*	808/404.5	6.55
PC_2-As-(OH) *	630	7.5
hm-PC_2-As-(OH)	660	6.9
PC_2-As-Cys *	733/367	8.00
hm-PC_2-As-Cys	763/382	7.30

As-$(GS)_3$*	994/497.5	12.43
GS-As- desGly-PC_2 *	862/ 431.5	16.92
GS-As- PC_2 *	919/460	17.55
desGly-PC_2-As-γEC	805/403	15.39
hm-PC_2-As-γEC	892/446	15.22 & 15.6
hm-GS-As-hm-PC_2	979/490	15.62
GS-As-hm-PC_2	949/475	16.48 & 16.95
As-PC_3*	844/422.5	20.39
As-hm-PC_3	874/437.5	19.55
As-desGly-PC_3	787	21.91
As-$(PC_2)_2$*	1151/576	22.44 & 23.74
Complexes identified in MA^{V**}exposed plants		
hm-GS-As-CH_3	426	6.91
GS-As-CH_3*	396	7.23
$(hm\text{-}GS)_2$-As-CH_3	763/382	13.69
hm-GS-As(-CH_3)-GS	733/367	14.61
hm-GS-As(-CH_3)- γEC	676/338.5	14.62
GS-As(-CH_3)- γEC	646/323.5	15.09
$(GS)_2$-As-CH_3	703/352	15.61
hm-PC_2-As-CH_3	658/329.5	19.46 & 20.18
PC_2-As-CH_3*	628/314.5	20.20 & 21.48
desGly-PC_2-As-CH_3	571/286	22.74

* Standards were available, **in all complexes As and MA are trivalent

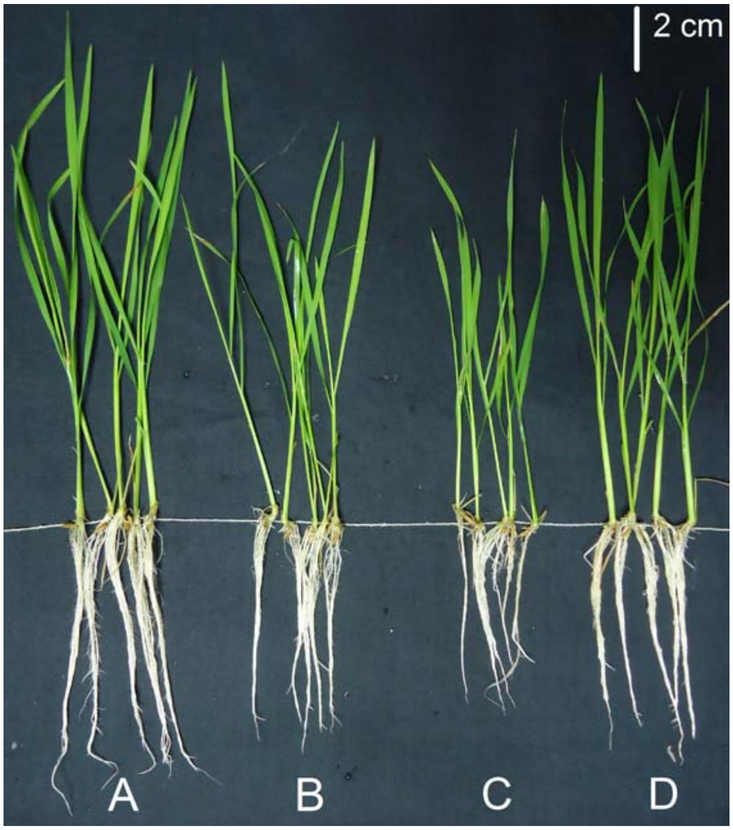

Supplementary Figure S1. Rice plants (*cv.* Triguna) exposed to different species of As. (A) Control plants, (B) Plants exposed to 10 µM As^{V}, (C) Plants exposed to 50 µM MA^{V} and (D) Plants exposed to 50 µM DMA^{V} .

Supplementary Method

Method for MA^{III} Synthesis and Identification. MA^{III} was synthesized by the method of Cullen et al., (1989)[53]. Briefly, methylarsonic acid sodium salt (MA^{V}) was dissolved in a small volume of warm deionized water and reduced by bubbling SO_2 through the solution for 20 min. After short-time boiling and fast cooling to 4°C the water was evaporated at 40°C with a TurboVap II (Zymark) to dryness. The residue was extracted by benzene and re-dissolved in water by a separating funnel. The aqueous phase was used for the ion chromatographic determination of MA^{III} using ICP-MS and ESI-Q-TOF-MS as detectors. The MA^{III} was identified by comparing the retention times of the arsenic-containing peaks obtained by ICP-MS at m/z 75 (As+) with the molecular ion peaks $[M+H]^+$ obtained by ESI-Q-TOF-MS. DMA^{V} was used as internal standard (**Supplementary Figure S2**).

Instrumental Setup

HPLC-ICP-MS/ESI-Q-TOF-MS consisting of an UPLC Series Infinity 1290 (Degasser, binary pump, thermostated autosampler) coupled with an ICP-MS 7500ce and Accurate Mass Q-TOF LC/MS 6530 in parallel (all Agilent Technologies, Santa Clara, USA) by a T-piece for splitting the mobile phase in 1:1 ratio. LC-ICP-MS/ESI-MS parameter used were: Column – IonPac AS7 and AG7 (10 µm, 4x250 mm and 4x50mm, Dionex, Sunnyvale, USA); Mobile phase - Eluent A: 0.04 mM HNO_3; Eluent B: 50 mM HNO_3; Gradient like ion chromatography; ESI-TOF-MS – positive polarity; Fragmentor voltage175 V; Capillary voltage 3500 V; Mass range 100-700 u; Gas temperature 325°C; Drying gas 10 L min^{-1}; Nebulizer pressure 20 psi; Sheath gas temperature 400°C; Sheath gas flow 12 L min^{-1}; Nozzle voltage 2000 V; ICP-MS – rf power 1600 W; Plasma gas flow 15 L min -1 (Ar); Carrier gas flow 0.6-0.7 L min-1; Sample depth 6 mm. The injection volume was 20 µL.

Supplementary Results

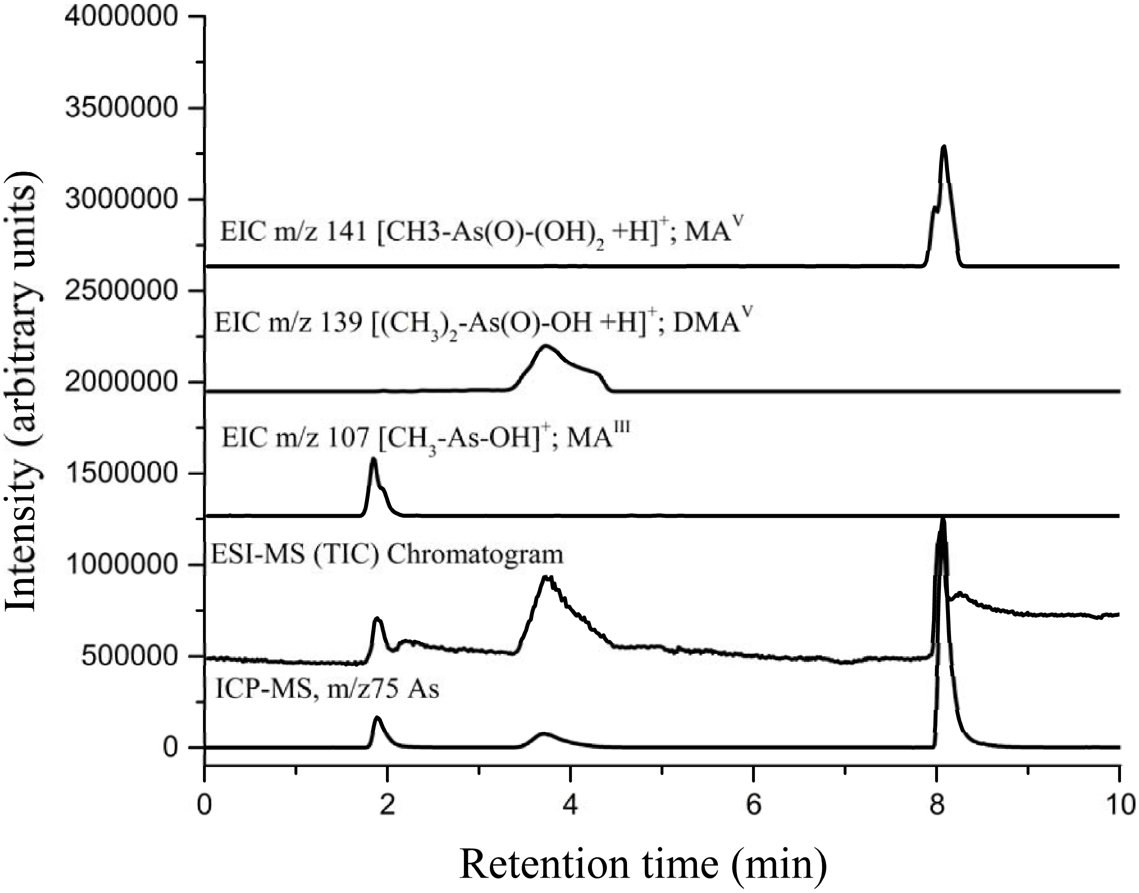

Supplementary Figure S2. Separation and identification of MA^{III} through HPLC-ICP-MS/ESI-Q-TOF-MS. MA^{III} was synthesized by the method of Cullen et al., (1989) and injected to HPLC online coupled to ICP-MS/ESI-Q-TOF-MS. ICP-MS trace of As (*m/z* 75) and ESI-Q-TOF-MS data (Total Ion Current and Extracted Ion Current chromatogram) are shown. DMA^{V} was used as internal standard.

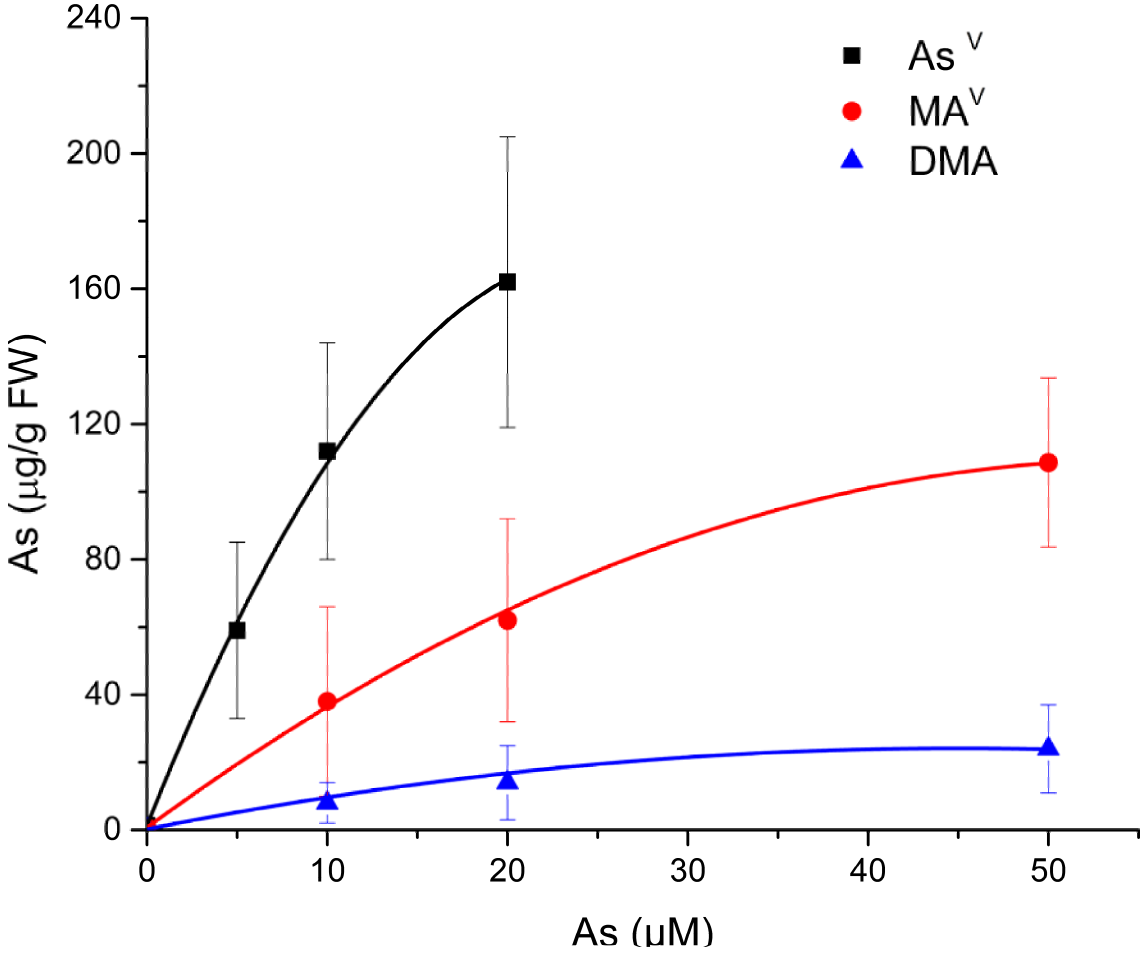

Supplementary Figure S3. Accumulation of total As (root + shoot) in rice exposed to various concentrations of As^V, MA^V and DMA^V for 7d. Values are mean ±SD, n=3.

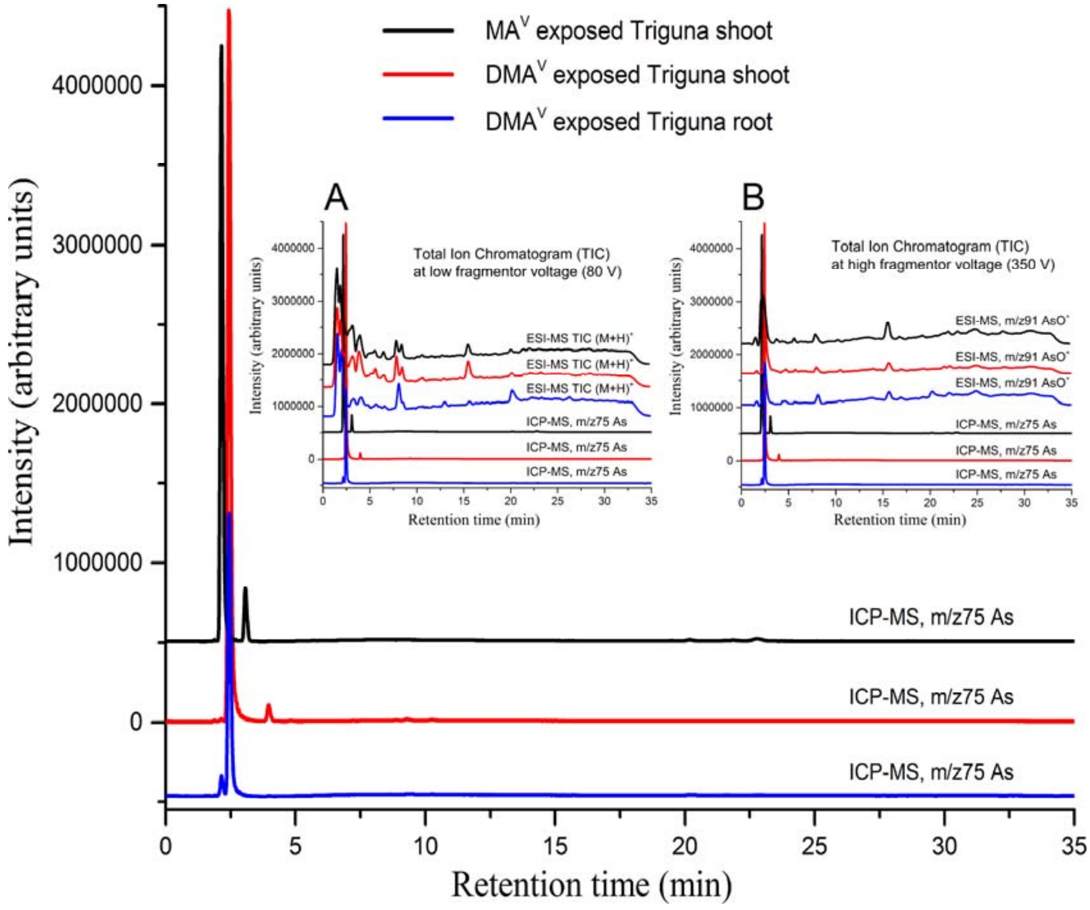

Supplementary Figure S4. RP-HPLC-ICP-MS/ESI-MS Chromatogram of As species in rice exposed to 50 µM MA^V or DMA^V. ICP-MS data (*m/z* 75 As) showing unidentified As species at 3.96 and 2.99 min respectively at DMA^V and MA^V exposed plant shoot. Inset (A) ESI-MS Total Ion Chromatogram (TIC) at low fragmentor voltage (80 V) showing $(M+H)^+$ and (B) ESI-MS TIC at high fragmentor voltage (350 V) showing the fragment AsO^+.

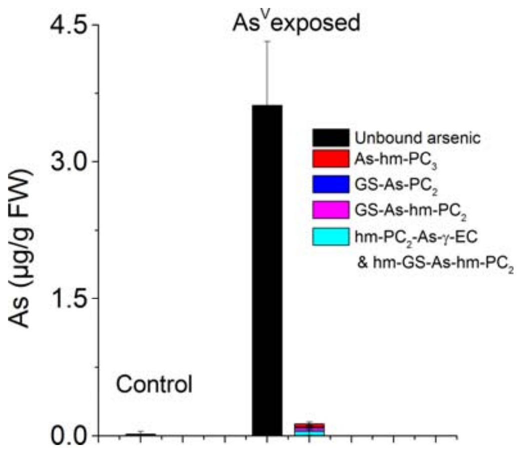

Supplementary Figure S5. Quantitative determination of thiol complexed As species analyzed through HPLC-ICP-MS/ESI-MS in fresh leaf extract of control and 10 µM As^V exposed rice plants. ICP-MS data (m/z 75) was used for the quantification. Values are mean ±SD, n=12. FW, Fresh weight.

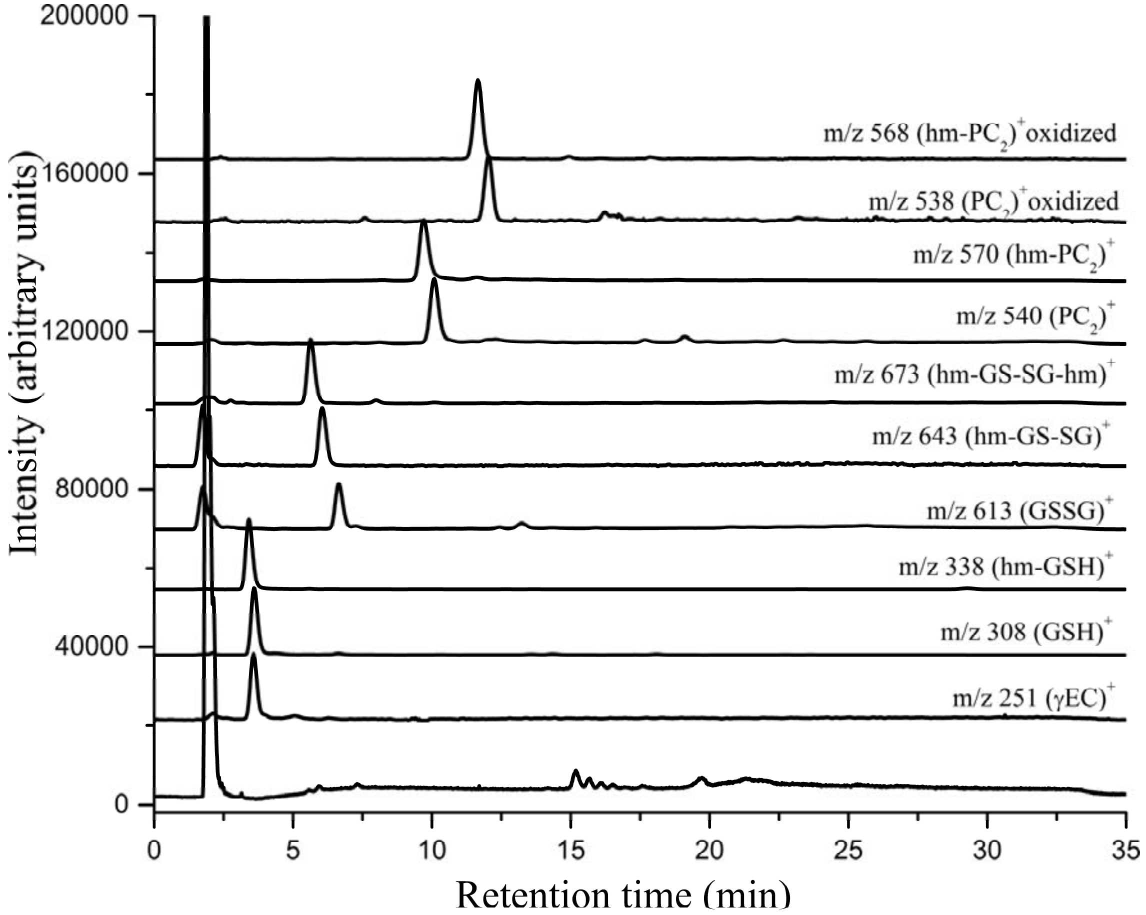

Supplementary Figure S6. Separation of thiols in shoot of rice exposed to As^{V}. ESI-MS data of thiols and ICP-MS trace of As (*m/z* 75).
